# IoT-Based Monitoring System Applied to Aeroponics Greenhouse

**DOI:** 10.3390/s22155646

**Published:** 2022-07-28

**Authors:** Hugo A. Méndez-Guzmán, José A. Padilla-Medina, Coral Martínez-Nolasco, Juan J. Martinez-Nolasco, Alejandro I. Barranco-Gutiérrez, Luis M. Contreras-Medina, Miguel Leon-Rodriguez

**Affiliations:** 1 Doctorado en Ciencias de la Ingeniería, Tecnológico Nacional de México/Instituto Tecnológico de Celaya, Celaya 38010, Mexico; d2003027@itcelaya.edu.mx (H.A.M.-G.); d1903010@itcelaya.edu.mx (C.M.-N.); 2 Departamento de Ingeniería Electrónica, Tecnológico Nacional de México/Instituto Tecnológico de Celaya, Celaya 38010, Mexico; alfredo.padilla@itcelaya.edu.mx (J.A.P.-M.); israel.barranco@itcelaya.edu.mx (A.I.B.-G.); 3 Departamento de Ingeniería Mecatrónica, Tecnológico Nacional de México/Instituto Tecnológico de Celaya, Celaya 38010, Mexico; 4 The Biosystems Engineering Group, Faculty of Engineering, Autonomous University of Queretaro—Campus Amazcala, El Marques, Querétaro 76140, Mexico; mcontreras.uaq@gmail.com; 5 Departamento de Ingeniería Robótica, Universidad Politécnica de Guanajuato, Campus Cortázar, Guanajuato 38496, Mexico; migueleon@upgto.edu.mx

**Keywords:** aeroponic, greenhouse, Internet of Things, irrigation, vapor pressure deficit, monitoring system, sensors

## Abstract

The inclusion of the Internet of Things (IoT) in greenhouses has become a fundamental tool for improving cultivation systems, offering information relevant to the greenhouse manager for decision making in search of optimum yield. This article presents a monitoring system applied to an aeroponic greenhouse based on an IoT architecture that provides user information on the status of the climatic variables and the appearance of the crop in addition to managing the irrigation timing and the frequency of visual inspection using an application developed for Android mobile devices called Aeroponics Monitor. The proposed IoT architecture consists of four layers: a device layer, fog layer, cloud layer and application layer. Once the information about the monitored variables is obtained by the sensors of the device layer, the fog layer processes it and transfers it to the Thingspeak and Firebase servers. In the cloud layer, Thingspeak analyzes the information from the variables monitored in the greenhouse through its IoT analytic tools to generate historical data and visualizations of their behavior, as well as an analysis of the system’s operating status. Firebase, on the other hand, is used as a database to store the results of the processing of the images taken in the fog layer for the supervision of the leaves and roots. The results of the analysis of the information of the monitored variables and of the processing of the images are presented in the developed app, with the objective of visualizing the state of the crop and to know the function of the monitoring system in the event of a possible lack of electricity or a service line failure in the fog layer and to avoid the loss of information. With the information about the temperature of the plant leaf and the relative humidity inside the greenhouse, the vapor pressure deficit (VPD) in the cloud layer is calculated; the VPD values are available on the Thingspeak server and in the developed app. Additionally, an analysis of the VPD is presented that demonstrates a water deficiency from the transplanting of the seedling to the cultivation chamber. The IoT architecture presented in this paper represents a potential tool for the study of aeroponic farming systems through IoT-assisted monitoring.

## 1. Introduction

The fundamental resources for traditional agriculture are farmland, soil and water. Conventional greenhouses require colossal infrastructures, having among their limitations uncontrolled environments, smaller farmland, plant diseases, soil degradation and a lack of resources, among others, leading to reduced crop production [1]. With the increasing demand for food, the role of agriculture demands the efficient use of resources and increased production. An option to improve farming systems focuses on stress monitoring and stress-causing factors in cultivation [2]. Consequently, various technologies have emerged for the conservation and use of water and energy, in addition to the benefits in increasing the production of multiple crops. Among the technologies that have had the most impact are wireless sensor networks (WSN), computing in the cloud, fog computing, embedded systems, big data, machine–machine (M2M) and human–machine communication (H2M), communication protocols, application programming interfaces (API), and advanced encryption standards (AES), as well as the use of geo-location, satellites and servers, among others [3].

In Mexico, it is necessary to increase agricultural production by at least 70% by 2050, increasing surface yield and reducing the amount of water consumed [4]. Recently, organizations such as the Secretaría de Agricultura y Desarrollo Rural, the Consejo Nacional de Ciencia y Tecnología (Conacyt) and the Instituto Nacional de Investigaciones Forestales, Agrícolas y Pecuarias (INIFAP) have agreed on the collaboration on and transfer of technology for cultivation in soil for semi-arid areas [5]. As time goes by, climate change and water scarcity drive national and international organizations to generate strategies to increase crop yield by making the most of water despite climatic adversities.

Aeroponics is a vertical cultivation method in which the plant grows suspended over a container, while a mist of nutrients is applied to the roots inside a protection chamber [6]. The nutrient solution is atomized on the root surface; for this, a pump is used that is in charge of the oxygen level in the root zone [7]. Since the nutrient solution is recirculated in the aeroponic cultivation technique, it is important to regularly measure and readjust the pH value and electrical conductivity (EC) to have a successful growth of the plant. If the pH and EC readings are not at the proper level, the cultivator should adjust the acidity and electroconductivity in the nutritious solution so that each crop has specific EC and pH levels; for example, the optimal EC and pH values for growing onions, cucumbers, carrots, lettuce, tomatoes and potatoes are between 1.5 to 2.5 ds m^−1^ and 5.5 to 6.5, respectively [8].

The pH and EC of a nutrient solution may be reduced due to environmental factors such as high temperatures causing water evaporation or the absorption of nutrients by plants [9]. In traditional soilless cultivation systems, such as hydroponics, where the EC and pH correction is performed manually, monitoring the temperature of the nutrient solution is sorely needed since the results of the conductivity and pH are directly influenced by this parameter [10]. The lifetime of the solution is of the utmost importance and will depend on timely adjustments made to the pH, electrical conductivity and water level. To exclude changes in the nutrient solution, the volume level in the storage tank must remain constant, replenishing the water absorbed by the plants and lost by evapotranspiration, otherwise, the concentration of the salts will change, affecting the healthy growth of the plants [11].

The practice of aeroponics is adaptable in areas where the soil is not suitable for growth of the plants, presenting advantages such as: a reduction in the cost of labor, savings in water of up to 98%, 60% in fertilizers and 10% in pesticides and herbicides, and maximizing the yield of the plant to between 45% and 75% compared to geoponic or hydroponic systems [8]. In comparison to cultivation techniques such as hydroponics and substrate, aeroponics has revealed a significant increment in root length, area, volume and network perimeter [12,13]. With respect to traditional agriculture, in aeroponics increments of 57%, 42% and 400% in the number of leaves, diameter of the leaves and root growth, respectively, considering a temperature inside the greenhouse between 8 °C and 44 °C and a relative humidity between 10% and 94%, have been observed [14].

On the other hand, in Mexico, 59.5% of the population has access to the Internet, which enables a high percentage of people to use and develop Internet of Things (IoT) technologies to improve the use of resources in agricultural crops [15]. Having described the above, assisted aeroponics with IoT tools appears to be a very attractive alternative to obtain crops of greater quantity and quality. One of the main challenges in aeroponics is to determine the atomization time and time interval in irrigation according to the needs of each plant [8]. To avoid a significant decrease in plant growth and reduction in crop yield, it is crucial to determine the threshold level to which each plant can be subjected under conditions of irrigation deficiency [16].

When a crop is under water stress, the root of the plant begins to turn dark, increasing its temperature. An indirect way to detect crop water needs is through changes in crop temperature and transpiration by measuring the crop water stress index (CWSI) [17], evapotranspiration (ET) [18], temperature difference between the environment and the leaf [19] and, recently, through the vapor pressure deficit (VPD) [20]. In arid and semi-arid regions, a crop is under water stress when the leaf temperature exceeds the air temperature from 4 °C to 6 °C; likewise, a crop does not present water stress when the temperature of the leaf is from 1 °C to 4 °C below the air temperature [21]. The water stress index can be calculated empirically by measuring the difference in temperature between the environment and the crop and normalizing it with respect to the differences between a humid crop due to high transpiration and a crop with low humidity due to the absence of moisture, or theoretically through the measurement of solar radiation, temperature and humidity in the environment and the temperature of the crop [22]. VPD and the change in the temperature difference with plant transpiration can be measured through leaf temperature, environmental temperature and relative humidity [23]. 

Evapotranspiration can be averaged through different methods, with the Penman—Monteith method being the most globally accepted; however, for this estimation the measurement of temperature, solar radiation, relative humidity, wind speed, atmospheric pressure and steam pressure saturation is required [24].

Related to the transpiration of a growing crop, the transpiration rate of a crop varies according to changes in atmospheric conditions, and one of the special factors is the VPD. A high VPD causes an increase in the rate of transpiration and therefore an excessive consumption of water and the photosynthetic limitation of the crop. A VPD above 1 kPa is potentially harmful, producing a reduction in stomatal conductance, making photosynthesis impossible and generating water stress even if the roots of the crop are well irrigated [20]. Reports of consecutive trials have recently been presented on crops on substrate applied to Salanova lettuce considering controlled environments in light intensity and temperature to modify the relative humidity and achieve a VPD of 0.7 kPa and 1.7 kPa during the growth cycle [20], following the methodology described in [25]. In these, it was found that the plant area was significantly greater in the crop developed with a low VPD. Other studies in tomato cultivation show that a low VPD moderates water stress, regulating the photosynthetic limitations of the plant and reducing the cumulative water consumption regardless of soil water status [26]. The fine regulation of and the decrease in VPD fluctuations are also crucial to produce a better crop growth since maintaining the photosynthetic performance of the plant leads to the leaf expansion being maintained and a higher yield being produced with high nutrition values [27]. In soil tomato cultivation systems, the applicability of IoT sensors was demonstrated to study the variations in the parameters of temperature, relative humidity and VPD [28]. Significant deviations from optimal climatic conditions due to imprecise heating and cooling systems used in commercial greenhouses were revealed in this study.

To address water stress, techniques based on temperature measurement from infrared sensors [23] and thermographic cameras have been used in the literature [29]. When using a thermal imager, a common technique for segmenting vegetative content is the simultaneous capture of visible spectrum (RGB) and multispectral images to determine the threshold of color components, generating a binary mask that will cover the thermal image and will show only the information of the vegetative components [29].

In aeroponics systems, the root development process is crucial for the plant’s growth performance. In order to optimize the plant’s aerial parts in aeroponic culture, the appropriate value definition of irrigation water pressure, droplet size and fogging interval is needed to improve the continuous water and nutrient availability [30,31]. Recently, non-invasive monitoring systems have been proposed for the study of vegetative growth parameters in roots and leaves developed in greenhouses [31]. 

Implementing and promoting the initial conditions for a healthy crop, using IoT-based technologies, requires monitoring devices with capabilities to acquire and analyze environmental, nutritional and crop data. In order to develop studies on soilless cultivation and determine better cultivation practices, monitoring of and controlling the main sensors and actuators involved in the aeroponic cultivation system are needed. The purpose of study in this paper is to determine the feasibility of integrating intelligent sensors, for the measurement of climatic conditions in the environment and crops as well as providing nutrient solutions, that allow favorable conditions for crop growth in aeroponic systems with IoT-based technologies. Considered variables for our proposal are temperature and relative humidity both in the greenhouse and in the plant leaf, VPD and luminosity, as well as the level, temperature, pH and EC in the nutrient solution tanks.

The proposed system presents the analysis of the information acquired by the sensors, the operating status of each of the elements incorporated in the platform using IoT analytics Thingspeak tools, as well as the initial preprocessing of the images acquired in the inspection from the leaf and root. Once the plant has been transplanted to the cultivation chamber, the plant must adapt to the defined irrigation scheme during the first week; therefore, the operation of each component incorporated in the proposed monitoring system is verified in the same period. 

For the experimentation, Batavia lettuce was chosen because its duration of the cultivation process is relatively short compared to other crops. The monitoring system incorporates the use of an application for mobile devices capable of showing records and visualizations from the analysis of information acquired by the built-in sensors and processed by the IoT analytics Thingspeak tools; in addition, the app records the measurements of the environmental conditions, the status and readjustment of the pH and EC of the nutrient solution, access to the irrigation controls and the recirculation and the mixer of the system. 

Additionally, the platform has a standard thermographic camera (8–14 μm); each of its captured images are transferred to the platform through an SMB server, which allows them to be viewed in the Aeroponics Monitor application. The Aeroponics Monitor was developed as a remote viewing and administration tool that allows users to modify the atomization time and the hours for the visual inspection of the crop, as well as the turning on and off of the sprinklers, the recirculation system and the mixture of nutrient solution, as well as maintenance tasks of the full system. The main advantages addressed by the proposal presented in this study are the remote administration of system actuators, the monitoring of each of the sensors incorporated in the greenhouse, the remote capture of images of the crops and the visualization of historical variables and data reports on the operation of each of the processes that are incorporated in the monitoring system, with all the above carried out through an application for Android mobile devices. 

This document is organized as follows. Section 2 presents a literature review that refers to the platforms and architectures used for the management of irrigation in crops. The configuration of the aeroponic system, description of the proposed monitoring system and the description of the Aeroponics Monitor application are presented in Section 3. Section 4 shows the results, while Section 5 presents the discussion and relevant contributions obtained in this work. Finally, in Section 6, the conclusions and future work are presented. The notation used for this article is described in Table 1.

## 2. Literature Review

### 2.1. Agriculture-Oriented IoT Architectures

There are multiple proposals in the literature referring to IoT architectures used in intelligent agriculture; among the most recent are AREThOU5A, AgriSens, IRRISENS, Agro-IoT, SWAMP and a proposed four-layered architecture by Kour in 2020, each referring to soil agriculture.

Kamienski et al. propose SWAMP, a generalized architecture oriented to the intelligent management of irrigation for agricultural systems on the ground incorporating different interconnection strategies between its elements to address the problems of communication in greenhouses on a large-scale using fog nodes in the soil with LoRaWAN routers. Its architecture is made up of five layers, which include the sending of information to end users, distribution models for irrigation, use of drones for vision inspection, data analysis models in the cloud fog nodes using as references the relative humidity of the soil, databases, security for data acquisition, sensors, actuators and weather reports, among others [32].

Based on the literature reviewed by Kour et al. from 2015 to 2020 and related to the advances and development of agriculture, the authors show, as a conceptual representation, a reference to IoT architecture for agriculture made up of six layers (Agro-IoT), including: a perception layer for sensors, actuators, wireless nodes, etc.; a network layer that encompasses the communication protocols, software middle layer, application layer for data analysis and prediction; a user layer where results are directed to farmers, experts in the area, the supply chain and industries. With the aim of monitoring low-scale greenhouses in real time, optimizing resource use, the early detection of diseases, identification of crop species, optimization of irrigation facilities and effective use of pesticides and fertilizers, the authors propose an IoT architecture for solar precision agriculture with four layers, including: a sensory layer, a network layer considering IoT nodes and base stations; a decision layer involving server services, workstations, work and knowledge base; an application layer involving how researchers, experts and farmers receive the information [33].

Filev et al. propose IRISENS, which is an architecture made up of five modules: the devices and network, kernel, external services, data management, intelligent services and user interface. In their proposal, they incorporate two models of microservices, one oriented to irrigation management based on the evapotranspiration of the crop and another for the collection of data from sensors and weather prediction services. Additionally, they incorporate data authentication between each module [34].

Boursians et al. suggest AREThOU5A a five-layer structure involving a physical layer, data link, network, authentication and application. Its main contribution is the integration of a weather forecast service to define better cultivation strategies, as well as the ability to use machine learning in data analysis and a solar charging system for RF communication devices located in the field [35].

Roy et al. propose Agrisens, described as an architecture of three layers: sensors and actuators, remote processing and service, and an application layer. The irrigation schemes are separated according to the life cycle of the crop in four stages considering the situations where the crop remains with water in the soil, irrigation under dry soil conditions and irrigation under wet soil conditions. Its structure offers the farmer the establishment of communication with the system through GSM messaging [36].

### 2.2. Irrigation Systems

Irrigation systems have been used in soil and soilless crops. Some of the most relevant ones are described below. In soil cultivation, there are different approaches to estimate irrigation needs in cultivation, highlighting the monitoring and control of the environmental temperature, soil moisture, evapotranspiration and water stress index. Authors such as González-Amarillo [37] and Fernández-Ahumada [38] use humidity and/or temperature measurement to turn on irrigation, ventilation and heating systems, while Mohamed [39] and Poyen [40] estimate the irrigation needs by performing the daily evapotranspiration measurement as reference. While Mohamed uses the Penman–Monteith method, Poyen includes as a feature of his system the automatic choice between the Hargreaves and Samni, Kharufa and Penman–Monteith methods according to climate type and geographical considerations. 

Jamroen et al. use a fuzzy controller to adjust the irrigation considering the variables of humidity and water stress index [22], while Lloret et al. perform irrigation by flooding, allowing automatic irrigation by measuring crop variables and climatic conditions, or remote activation using a mobile app [41].

In soilless farming, Puengsungwan et al. propose a method to determine root stress in relation to the difference in environmental leaf temperature and light intensity in hydroponic crops considering environmental temperature control between 28 and 32 °C. In their proposal, the patterns obtained are separated by regions considering a normal root, with stress and a rotting root. Using IoT technologies, they reduce the response time of the system from 5 min to less than 60 s and increase the efficiency in detection from 85% to 95% in comparison with a method based on identifying the reduction in the leaf area of the crop by means of the Easy Leaf Area app [23].

In aeroponic crops, the main variables monitored to manage the irrigation systems are the temperature and humidity inside the greenhouse. Lucero et al., monitored the temperature and humidity inside a greenhouse used for aeroponic cultivation, establishing the times of irrigation for the day and night under three stages according to the days of production. In their proposal, when the temperature exceeds 35 °C, the irrigation period is reduced. In their study, they compared a crop of 21 lettuces showing that an aeroponic system outperforms the traditional crop in soil in terms of the number of leaves, average diameter of the leaves and average length of the root by 57%, 42% and 40%, respectively [14]. Jamhari et al. present the control of the relative temperature and humidity in the root chamber, in which they turn on a Peltier cell that cools the nutrient solution maintaining a range from 25 to 29 °C in the chamber, while the relative humidity is controlled between 50% and 70% through an ultrasonic humidifier and a fan [42]. Gour et al. propose the use of a central processor with an interface between sensors, actuators and machine learning tools to automate the cultivation process [43]. On the other hand, some proposals consider the use of IoT architectures for the monitoring and control of devices. Aiming to implement a vertical cultivation chamber to care for crops from germination to harvest, Belista et al. present the design of a vertical cultivation chamber distributed in master and slave modules. The master module controls the nutrient containers, the water-cooling system, an evaporative fan and a power supply, while the slave modules control the pumps that transport the nutrients to the crop. The IoT architecture that is presented to manipulate the weather control systems, the aeroponic irrigation and the mixing chamber for nutrients is based on a central controller, which reads and writes commands to acquire information from the sensors and thus manipulates the system actuators. The information acquired is stored locally, where the user can access the information through an application for mobile devices that is linked to the central controller through a SAMBA server [44]. 

According to the literature, there are a wide variety of IoT architectures according to the functionality and elements that each author defines between communication, the database, devices integrated into the network, security, services or microservices and their relationship with the end user. Similarly, among the various forms of cultivation, the variables in common are temperature, humidity, transpiration and luminosity.

The previously mentioned proposals present connectivity schemes mainly oriented to the acquisition of information from climate sensors for weather conditions, control of actuators for the adjustment of weather conditions and irrigation schemes, and user access to the systems to supervise the information collected and to be able to modify the irrigation strategies; nevertheless, they do not contemplate the study of climatic variables for the analysis of the VPD and the appearance of water stress.

To approach the study of water stress in relation to the frequency and time of irrigation, as well as the climatic variables in the environment and cultivation in a practical way in aeroponic systems, the system proposed in this study integrates features such as crop temperature measurement, irrigation programming, automation through microservices, the use of a central processor and the integration of a SAMBA server as addressed by Puengsungwan, Lucero, Loret, Gour and Belista, respectively. However, our proposal has sensory elements, processing, administration, storage and remote access through a 4-layer IoT architecture with the flexibility of incorporating more sensory elements, processing and decision-making capabilities through the fog layer, and system administration functions in the user layer for further studies to modify the functional structure of the proposed IoT architecture. The IoT architecture presented consists of an end devices layer, fog layer, cloud layer and application layer. Among the main features incorporated in our proposal are the following:

1. The grouping of electronic sensors and vision systems with end devices.
2. The administration of end devices through independent services for reading sensors, and the remote activation of actuators and acquisition systems for standard WEB, USB and thermographic cameras using a SAMBA server. Additionally, it has an alert service via SMTP in case of failure of the other services. All of the above is through the fog layer.
3. A local and cloud database. The local database is dedicated to the storage of RGB top, side and root images, as well as thermographic images in the root. The database in the cloud is used for the storage of time-series data generated by the electronic sensors, in addition to the status of the sensors and actuators, the operating status of the services of the system, as well as the storage of the last result of the image processing.
4. The implementation of an Android application for the manual registration of the variables related to the nutrient solution, environmental conditions, crop yield, historical display, data analysis reports, as well as the remote acquisition of images, activation of actuators and modification of irrigation parameters.

Table 2 highlights the main characteristics of each architecture with respect to our proposal.

## 3. Monitoring System

### 3.1. Aeroponic System Setup

**Figure 1:**
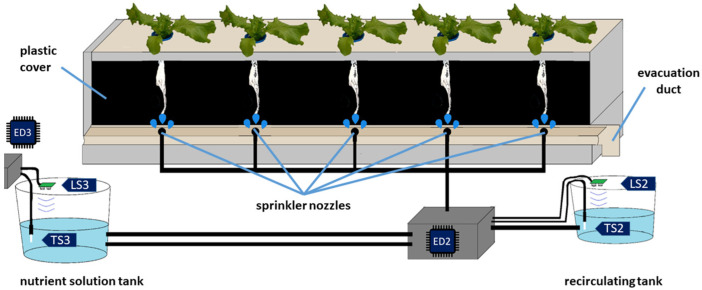
Aeroponic growing chamber.

The aeroponic growing chamber is formed by a structure with an upper base of 2000 × 300 mm and holes of 55 mm, with a spacing of 30 mm for the placement of 5 lettuces. Inside the growth chamber, a 2.5-inch duct was placed along the structure, where 5 sprinkler nozzles as well as a collector duct for the excess nutrient solution that returns from the crop (Figure 1). The nutritive solution tanks used by the irrigation and recirculation system have a capacity of 83.5 and 21.5 L; in both tanks the level and temperature are monitored using the HC-SR04 (LS2 and LS3) and DS18B20 (TS2 and TS3) sensors. Two R385 diaphragm pumps are used to distribute and recover the nebulized nutrient solution from the roots. When the level of the recirculation tank exceeds 16 L, a pump is activated to transport the recovered nutrient solution to the main tank, later using a submersible pump with a capacity of 160 L/h to mix the solution inside the main tank.

**Figure 2:**
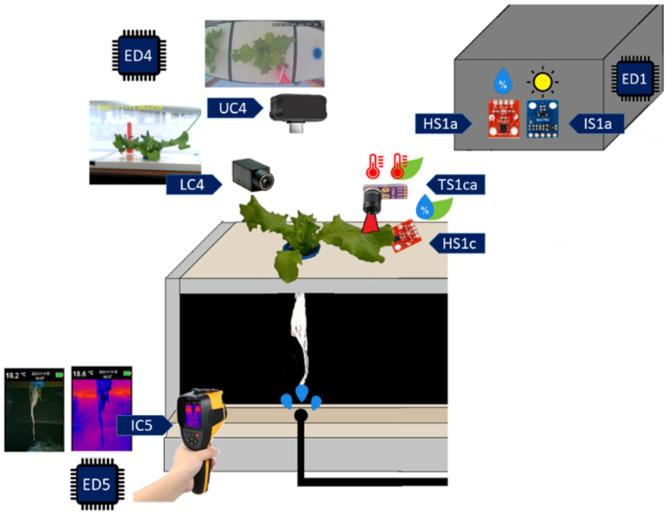
Location of sensors and visual inspection system.

For the measurement of the environmental temperature, the leaf temperature and the relative humidity of the leaf, the sensors MLX90614 (TS1ca) and HTU21D (HS1c) are used; these are located on the upper part of the lettuce leaf, while for the variables of relative humidity of the environment, luminous sensors HTU21D (HS1a) and BH1750 (IS1a) are used, which are located above the aeroponic chamber. For crop growth recording regarding the height of the plant and leaf area, two cameras with acquisition capacities over the visible spectrum are considered, a webcam (LC4) and an IP camera (UC4), both located on the structure of the growth chamber, one on the side and the other on the top. For the registration of the colorimetry, the length of the root and the temperature in the root chamber, RGB and thermographic images are captured by a standard thermographic camera (IC5) and sent to the fog layer by connecting the thermographic camera to a mobile device or computer through an SMB user. Figure 2 shows the location of the sensory elements for the measurement of the previously described variables, as well as the visual inspection systems.

### 3.2. Monitoring System Proposal

**Figure 3:**
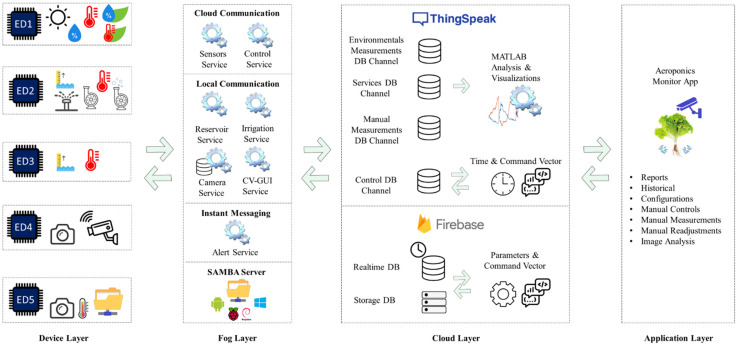
IoT architecture of the monitoring system.

The proposed monitoring system is based on a four-layer IoT architecture: device layer, fog layer, cloud layer and application layer (Figure 3). In Figure 1 and Figure 2, the end devices incorporated in the device layer of Figure 3 are labeled. End device 1 (ED1) integrates the sensors used for the measurement of temperature and luminosity in the greenhouse, as well as with the leaf temperature and humidity sensors. End device 2 (ED2) integrates the level and temperature sensors of the recirculating solution tank, as well as the pump activation system for the stages of irrigation, recirculation and mixing between the recovered nutrient solution and that stored in the main tank. End device 3 (ED3) integrates sensors for level and temperature measurement from the main solution tank. End device 4 (ED4) integrates the cameras used for the registration of the growth of the crop. Finally, end device 5 (ED5) is made up of a thermographic camera and a mobile device to send the information to the fog layer. The device layer consists of five end devices, ED1, ED2 and ED3 are made up of NodeMCU development boards based on Web Server, ED4 has a standard USB camera and an IP camera, both managed by the fog layer through a Raspberry development board, as long as the device has a standard USB thermal imager connected to a mobile device based on SMB. The information acquired by each end device is transmitted to the fog layer.

The fog layer is made up of a set of microservices in charge of managing the information locally or remotely through one of several servers in the cloud. Locally, 4 services are executed independently: irrigation service in charge of turning the sprinkler system on and off according to the irrigation frequency and the day and night switch-on time programmed by the user; reservoir service responsible for monitoring the level of the secondary tank and generating the recirculation of the nutrient solution to the main tank; the camera service in charge of taking screenshots of the plant from the side and top view according to the schedule programmed by the user, generating a local database; likewise, it is in charge of processing the images coming from all the cameras installed in the system; CV-GUI Services, the remote user interface used to manage system settings and status.

On the other hand, the sensor service, control service and alert service microservices manage the exchange of information between the sensors, local microservices and the Thingspeak server. The sensor service is responsible for collecting information from each one of the sensors of the environmental variables, the nutrient solution, as well as the state of execution of the irrigation systems, the recirculation of the nutrient solution and the activating of the cameras installed to upload the data to the cloud. The control service has the function of scanning the server’s control data channel before the start of new configurations for the irrigation system and the camera system, as well as the remote activation of the sprinkler nozzles, the recirculating stage and the nutrient solution mixer. The alert services are in charge of monitoring the operation of all the services; in case one of them is interrupted by a failure, it sends an alert via SMTP. In addition to the microservices, the fog layer integrates an SMB server, which allows connectivity between the standard thermographic camera and the image database through its USB connection to an Android, Windows or Linux device, and this in turn as an SMB user. 

The cloud layer is made up of the data storage and analytics services built into the Thingspeak and Firebase platforms. Thingspeak is used as a database with a time-series format, as well as for data analysis and visualization of results through its IoT analytics tools. For this investigation, four Thingspeak channels are used for: the administration of the variables of the environmental temperature, the temperature in the leaf, the relative humidity in the environment and the leaf, the level and the temperature of the recirculation deposit, as well as light intensity (environmental measurements channel); administration of the variables related to the level and temperature of the main nutrient solution tank and the operating status of the chambers, the irrigation system and the recirculating stage (services channel); storage of variables ambient temperature, relative humidity, crop temperature, pH, EC, fresh weight, length and width of the lettuce leaf, all of them implemented manually using standard instruments and recorded through the app generated in this study (manual measurements channel); management of requests from the end user to download or update administration of requests for the irrigation times and camera triggers, as well as remotely activating the irrigation system, the recirculating stage as well as the nutrient solution mixer (control channel). Firebase, on the other hand, has a real-time database stored in JSON format, as well as a database for file storage. The use of Firebase is intended for the configuration of the parameters for the processing of the images, as well as the storage of the images processed by the fog layer, allowing the platform to request specific image processing services between the application developed in this research and the fog layer.

Finally, the application layer is an Android app called “Aeroponics Monitor”, which, through HTTP requests, makes requests to the Thingspeak server for the generation of comparative or analytical reports between variables, performance reports of installed sensors and communication with the platform, historical data by variable, access to the configuration of the irrigation systems and the triggering of the image capture devices as well as the manual control of actuators through remote access, the measurement of the cultivation parameters and the registration of the amount of nitric acid and solution A and B dissolved in the nutrient solution to readjust the pH and EC ranges. Likewise, it has access through Firebase to the latest results of the processing of the images generated by the fog layer.

### 3.3. Aeroponics Monitor App

**Figure 4:**
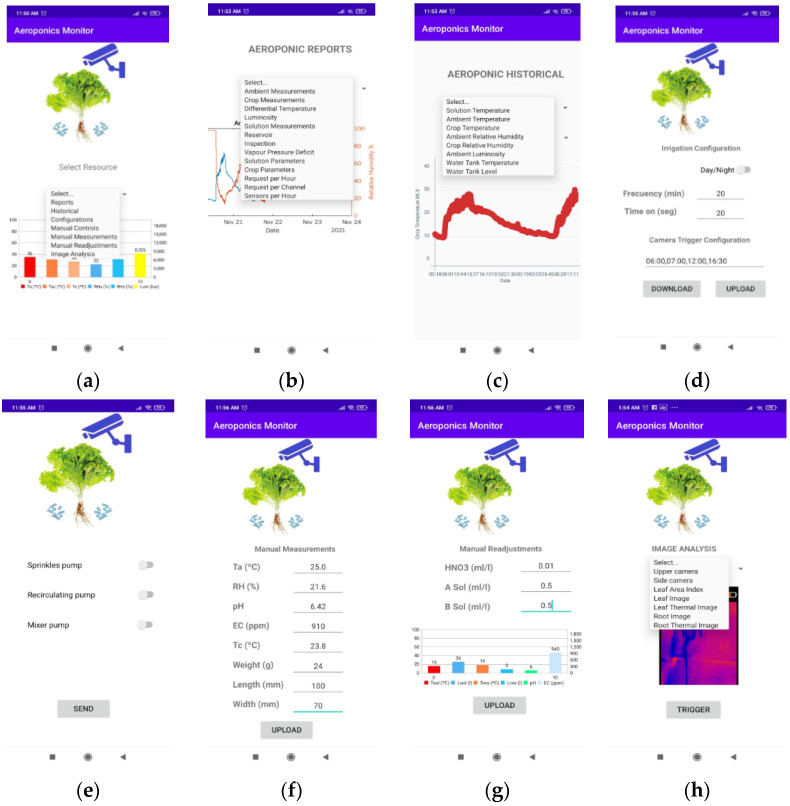
Windows of Aeroponics Monitor. (**a**) Initial window; (**b**) report window; (**c**) record window; (**d**) settings window; (**e**) manual controls window; (**f**) manual measurements window; (**g**) manual readjust window; (**h**) image analysis window.

The Aeroponics Monitor is an application developed at Android Studio and registered in INDAUTOR Mexico under the registration number 03-201-10514230-01; this application is operated by users to visualize the behavior of the variables and the operating status of the system, and control the actions of the aeroponic system actuators in a remote way. The Aeroponics Monitor contains eight main windows: the home window (Figure 4a), which allows a quick view of the status of the environmental variables of the culture system, accessing through a menu section of reports, records, configurations, manual controls, manual measurements, manual readjustment and image analysis. Each one of them is described below.

#### 3.3.1. The Report Window

The report window requests a visualization of IoT analytics to Thingspeak to show the behavior of the last three days of a specific variable or the relationship between two or more variables to generate a report on the operation status of a component of the system (Figure 4b). The relationship between the variables and the reports that display this sale is shown in Table 3.

#### 3.3.2. Record Window

The record window allows the view of the variables of the aeroponic system individually, showing an analysis of the data stored for one hour, 12 h, one day and up to three days by making an HTTP request to the server. Among the variables monitored in this section are the temperature of the nutrient solution, the environmental temperature, the crop temperature, the relative humidity of the environment, the relative humidity of the crop, luminosity, temperature of the recirculation tank and level of the recirculation tank (Figure 4c).

#### 3.3.3. Settings Window

The settings window gives users access to modify the parameters for activation of the image acquisition system, as well as the frequency and time of ignition (day/night) of the irrigation system (Figure 4d).

#### 3.3.4. Manual Controls Window

The window for manual controls allows a link to be established remotely with the irrigation system. Its operation is based on downloading the latest state of each actuator to show it on the app’s switches, as well as updating each state when the “SEND” button is pressed, so that the control service executes the orders in the greenhouse (Figure 4e).

#### 3.3.5. Manual Measurements Window

Through this window, the user makes a manual record of the evolution of crop parameters, such as fresh weight, length, width and temperature of the lettuce leaf; the evolution of the environmental variables considering the temperature and the relative humidity in the environment; the evolution of the parameters in the nutritive solution considering the pH and EC. All these parameters are stored in the server for the generation of the record and reports (Figure 4f).

#### 3.3.6. Manual Readjust Window

The manual readjust window allows the user to check the temperature and level of each tank in the aeroponic system, as well as a quick reading of the latest pH and EC value (Figure 4g). To readjust the pH and EC value, the total evacuation of the recirculation tank must be considered through the manual control window to later read the number of liters in the main tank and record the amount of nitric acid used to lower the pH, as well as the amount of solution A and B to compensate for the quantity of salts dissolved in the nutrient solution.

#### 3.3.7. Image Analysis Window

The image analysis window allows viewing the latest data capture taken by the image acquisition systems of the monitoring system. Among the results that allow visualization of the platform are the lateral image, the upper image, the processing of the leaf area index (LAI), as well as the latest RGB images and IR of leaf and root obtained by the thermal camera (Figure 4h).

### 3.4. Data Management

Among the main challenges faced in IoT applications are data offloading, heterogeneity and big data [45]. To reduce the latency between the IoT sensors described in the proposal and the Thingspeak server, sensor service makes consecutive requests via HTTP protocol to each of the end devices, collecting and grouping the information, from climate and nutrient solution sensors, in a single package and registering it on the server. Between each request from sensor service to server, 30 s waiting interval is taken.

To address the heterogeneity of the data, sensor service operates sequentially, while the services oriented towards image capture and irrigation and recirculation services operate in parallel through the camera service, irrigation service and reservoir service, respectively. Each service in operation generates a binary log file (npy file), storing the latest state of sensors and actuators, through which it shares the information with other services running on the fog layer.

Regarding the amount of data that can be generated between numerical records and images through IoT devices, numerical records are stored in Thingspeak with a maximum of 2880 requests per day out of 8200 possible, while the image records from the side and upper view of the plant are directly stored in the SD memory on the fog layer. The number of images generated depends on times scheduled for image acquisition (9/day/camera in this experiment).

### 3.5. Remote Operation

**Figure 5:**
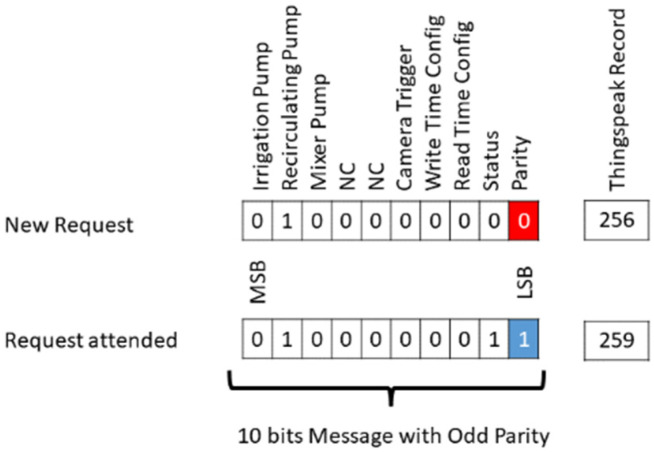
Command vector for remote operation from App.

Remote operation of the aeroponic system is achieved from reading (continuous) and writing (on demand) of the control’s data channel (command vector), which hosts the latest status of the system’s irrigation, recirculation and mixer actuators, as well as the command states that trigger the image capture system, writing or reading of configurations referring to the interval and frequency of irrigation (Figure 5). Each of these states is encoded and decoded in the communication process between fog layer and the app.

Command vector is a message encoded in 10 bits, a bit for the activation or deactivation (1 or 0) of any actuator or service hosted in the fog layer, followed by a status bit to detect if the request has been attended or not (1 or 0) by the fog layer, ending with a parity bit to avoid errors in the communication between the app and fog layer. Response messages between the communication are stored in numerical form in Thingspeak control’s data channel.

### 3.6. Cost of Using the Platform

To establish the minimum operating conditions of the proposed architecture, free licenses for Thingspeak and Firebase servers are used. Thingspeak under a free license allows a message limit of approximately 3 million/year or 8200 messages/day with an update rate of 15 s. Likewise, it allows us to use 4 channels with 8 fields for numerical data. Currently, the development of this work requires 27 of the 32 available fields. In case of requiring more fields than assigned, a student, home, standard or academic license can be obtained, specifying the number of channels and update rate (1 s as minimum) with a cost from USD 79.00 for 33 million/year/units [46]. Among the services offered by Firebase subscription are real-time database and cloud storage, which are used in this project in its free mode (Spark) to store the most recent results of image processing according to the schedule assigned by camera service or downloading them by request from the app. Firebase in these services allows storage of 1 GB and 5 GB, respectively. In the case of the real-time database, it allows downloads of up to 10 GB/month and in the case of cloud storage a bandwidth of 1 GB/day. For higher storage, bandwidth or additional features, Firebase offers a pay as you go (Blaze) plan and a cost calculator in its website [47].

### 3.7. Improvements Using a Fog Layer

In cloud-based IoT environments, latency is often high due to the distance between IoT devices and the cloud, this increases cloud response time. As the number of IoT devices increases, the cloud cannot support the real-time demands of these [48]. According to Thingspeak, for each write request attended, a message is consumed [49]. By using a fog layer in our proposal, it allows grouping the information in packets of 8 numerical data, uploading them to the server using only a single request per channel. This involves handling writing to the server of up to 32 numeric variables with a single wait interval. On the other hand, Firebase has the Firebase ML service, which has models with the ability to recognize text, image labeling, object detection and tracking, among others; however, it is limited to the use of TensorFlow Lite models [50]. Using a fog layer as a central processing element allows the execution of multiple vision algorithms without dependence on external service or additional usage cost. In consideration of the use of Firebase only as storage for image processing results, the bandwidth used to download data from the server to the app implies only 150.05 kB/request/user.

## 4. Results

In order to test the functionality of the monitoring system, a Batavia lettuce seedling was subjected to observation in the aeroponic crop chamber for 7 days from its transplant, placed in the main tank with 50 L of prepared nutritive solution with 5 mL/l of solution A and 5 mL solution B, with pH initial 6.0 y EC 915 ppm. From the transplant, the platform was set up to irrigate the seedling every 24 min day/night with an activation time of 30 s; likewise, the lateral and frontal image acquisition system was set up to perform shots at 6:00, 7:00, 8:00, 11:00, 12:00, 13:00, 17:30, 18:30 and 19:30 h. RGB and thermal images on the root were captured between 14:00 and 14:30 h. The status of climatic and crop variables was acquired every 30 s. The pH level and the electrical conductivity were taken manually daily, as well as the manual measurement of the dimensions of the lettuce leaf and its fresh weight. 

The results of this study have been classified into two sections: the first section refers to the registration of the monitored variables and the reports generated by the IoT analytics in the Thingspeak server, as well as the registration of images stored in the fog layer; the second section focuses on the analysis of the environmental variables and the findings made by contrasting them with the RGB and thermographic images captured.

### 4.1. Record of Monitored Variables and Image Acquisition System

**Figure 6:**
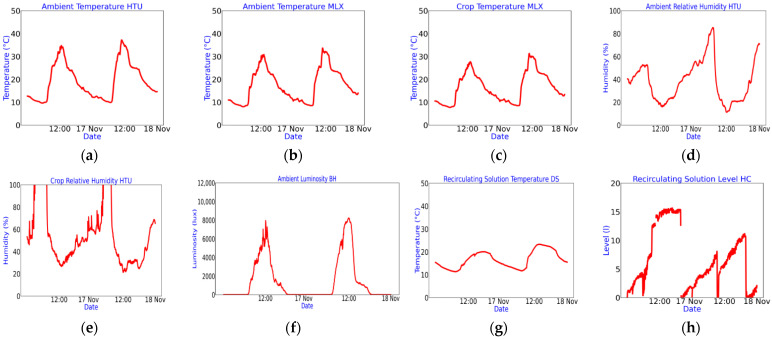
Recording of environmental variables in a Thingspeak channel. (**a**) Environmental temperature of the HTU21D sensor; (**b**) environmental temperature of the MLX90614 sensor; (**c**) crop temperature; (**d**) relative humidity; (**e**) relative humidity in the crop; (**f**) luminosity; (**g**) temperature of the recirculating solution; (**h**) level of recirculating solution.

Figure 6 shows the recording of environmental variables in a Thingspeak channel; the following are recorded: (a) environmental temperature of the HTU21D sensor, (b) environmental temperature of the MLX90614 sensor, (c) crop temperature, (d) relative humidity, (e) relative humidity in the crop, (f) luminosity, (g) temperature of the recirculating solution and (h) level of recirculating solution.

**Figure 7:**
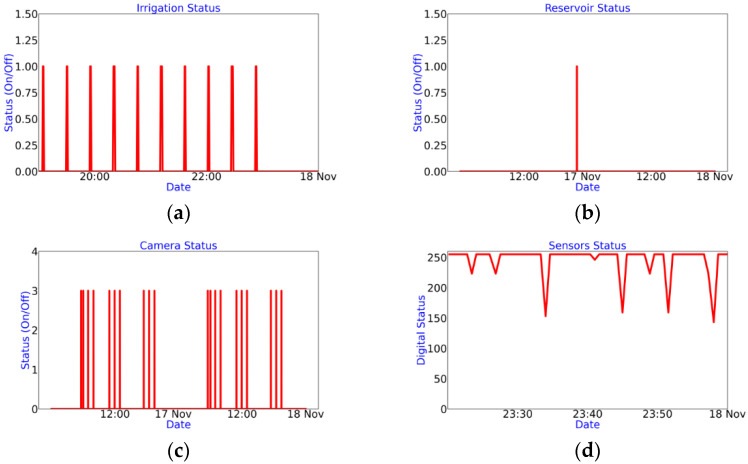
Status of critical microservices. (**a**) Irrigation system activation log; (**b**) recirculation system activation log; (**c**) triggers of the image acquisition system; (**d**) recording of successful measurements made by the sensors of the environmental variables channel.

A second Thingspeak channel is used for reporting the status of critical microservices (Figure 7), such as: (a) the irrigation system activation log, (b) the recirculation pump trigger, (c) trigger of the image acquisition system and (d) the recording of successful measurements by the sensors of the environmental variables channel. These indicators are vital to determine the functionality and failure detection of the electronic systems connected to the architecture: failure events in the irrigation system, recirculation and image acquisition systems, are visually appreciated by the absence of shown impulses in Figure 7a–c, respectively; electrical failure of any electronic sensor, is visually appreciated by the decrease in amplitude in (d). Since this is a numerical register that varies from 0 to 255 and is generated as a union of 8 bits, each bit is an indicator of the correct reading of each electronic sensor incorporated in the architecture, 1 or 0 if the reading is successful or not, respectively. If a bit frequently remains on 0, this implies a communication problem with the sensor and its possible need for replacement.

**Figure 8:**
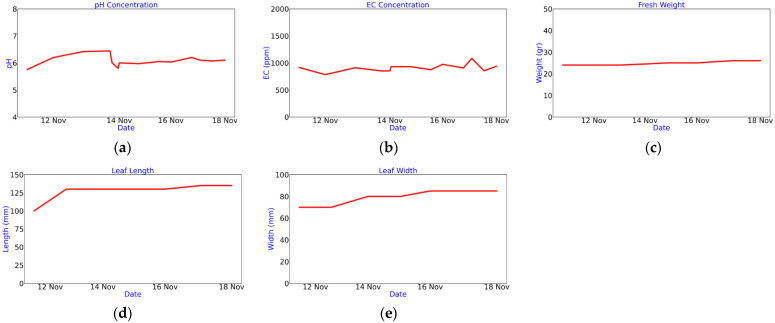
Manual register of variables in nutrient solution and crop. (**a**) pH; (**b**) EC; (**c**) fresh weight; (**d**) leaf length; (**e**) leaf width.

In a third channel of Thingspeak, the measurements made regarding the nutrient solution and the evolution of the crop are saved (Figure 8), considering the: (a) pH, (b) EC, (c) fresh weight, (d) length and (e) width of the leaf.

**Figure 9:**
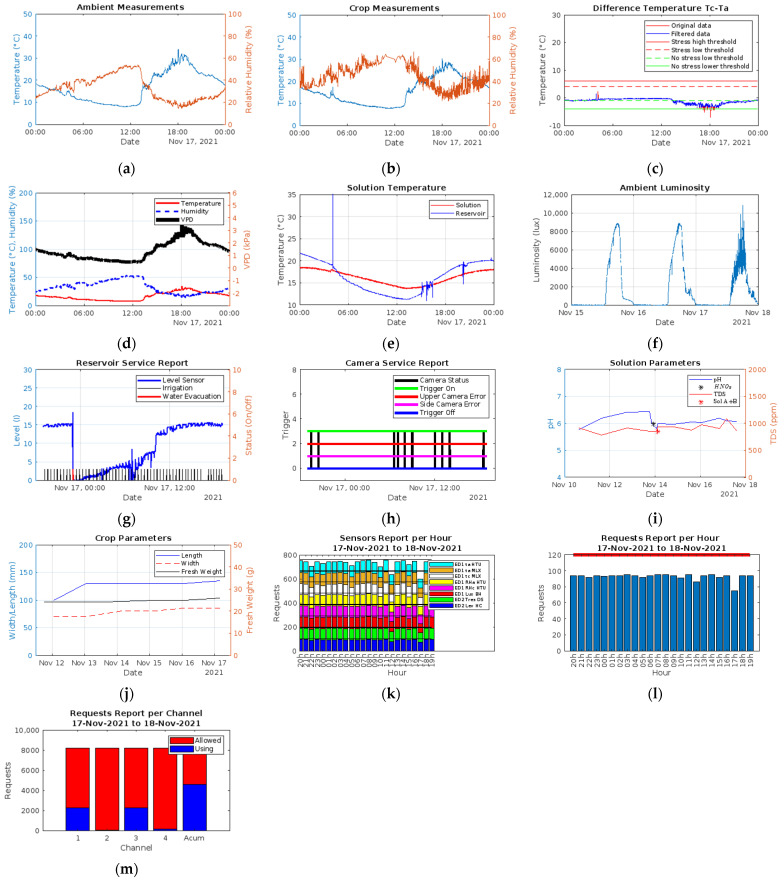
Reports generated by Thingspeak using MATLAB visualizations. (**a**) Temperature measurement in solution tanks; (**b**) measurement of temperature and relative humidity in crop; (**c**) crop minus environment; (**d**) vapor pressure deficit; (**e**) temperature in tanks; (**f**) ambient luminosity; (**g**) report of the level of the recirculation tank and performance of irrigation and recirculation systems; (**h**) operation state of the imaging system; (**i**) parameters in nutrient solution pH and EC; (**j**) crop growth parameters; (**k**) report of successful readings on sensors per hour; (**l**) reporting of requests sent to the server by hour; (**m**) reporting of requests sent to the server by channel.

Figure 9 shows each of the visualizations generated from Table 3; visualizations are obtained by request from the Aeroponics Monitor app to Thingspeak: (a) relative temperature and humidity in the environment, (b) relative temperature and humidity in the crop, (c) temperature difference between crop and environment and its classification regarding the detection of water stress, (d) temperature and humidity against VPD, (e) temperature in the recirculating and main tank, (f) luminosity, (g) monitoring of level, irrigation and recirculation system activation, (h) image acquisition system activation and its classification against failures, (i) pH, EC indicators of nutrient solution readjustment, (j) crop evolution, (k) report of successful sensor readings/h, (l) requests sent to the server per hour and (m) total requests sent to the server per channel.

**Figure 10:**
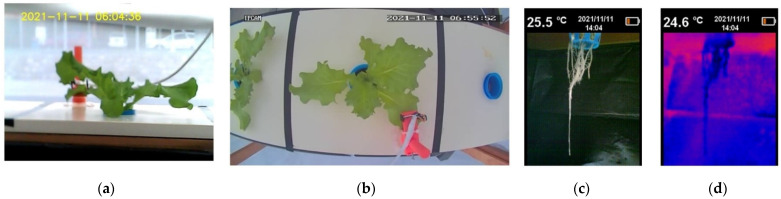
Images acquired by the proposed intelligent monitoring system. (**a**) Lateral view of the crop; (**b**) top view of the crop; (**c**) RGB image of the root; (**d**) thermographic image of the root.

Graphs (g), (h), (k), (l) and (m) allow you to quickly review the operating status of the platform in the app; (g) shows the evolution of the level of the solution in the recirculating tank from the activation of irrigation and the recirculation pump. Among the main failures that can be detected from its review are sprinkler obstruction (increase in period or stagnation), irrigation service failure or recirculation (absence of impulse) and level sensor failure (frequent reading at 0); (h) shows the state of the image acquisition system, or normal operation, and is represented by the appearance of the pulse amplitude of 3, while a stagnation in 2 or 1 determines a flaw in the upper and frontal chamber, respectively, or the absence of impulse, the failure in the camera service; (k) and (l) are complementary indicators; the absence of (k) denotes a failure on the sensor service, while the absence of (l) denotes an internet connection failure (l); (m) shows the number of requests sent to the server per channel as a whole, avoiding crossing the limit of 8200 requests sent per day. Finally, Figure 10 shows an image acquired by the image acquisition system incorporated in the proposed system.

### 4.2. Analysis of the Environmental Variables and Image Analysis

For the analysis of the environmental variables, the database obtained in the period between 1 and 17 November 2021 was downloaded from Thingspeak. To define water deficiencies due to the modification of plant transpiration with reference to climatic variables in the greenhouse, VPD was calculated. 

VPD is defined as the difference between the water vapor pressure at saturation (*P_sat_*) and the actual water vapor pressure at the temperature of the greenhouse [51]. *P_sat_* can be calculated from Allen, 1998 [52] as follows

(1) $$P_{sat}\left(T\right)=0.6108e^{\;\;\;\frac{17.27\;T}{T+237.3}}$$

with

(2) $$VPD=P_{sat}\left(T\right)-\left(\frac{RH}{100}\right)P_{sat}\left(T\right)$$

where substitution of the leaf temperature and greenhouse temperature in (1) results as

(3) $$VPD=0.6108e^{\;\;\;\frac{17.27\;T_{c}}{T_{c}+237.3}}-\left(\frac{RH}{100}\right)0.6108e^{\;\;\;\frac{17.27\;T_{a}}{T_{a}+237.3}}$$

or expressed as a base 10 exponential form [23,53], approximately as

(4) $$VPD=610.7\left[10^{\frac{7.5T_{c}}{T_{c}+237.3}}-\left(\frac{RH}{100}\right)10^{\frac{7.5T_{a}}{T_{a}+237.3}}\right]$$

**Figure 11:**
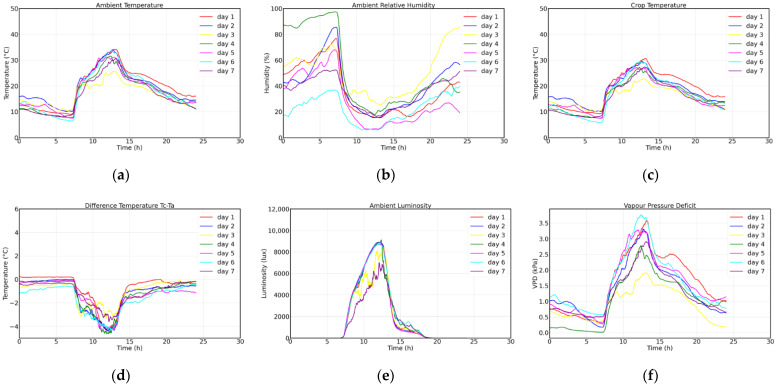
Registration of environmental variables and markers of water stress. (**a**) Ambient temperature; (**b**) ambient relative humidity; (**c**) crop temperature; (**d**) crop—ambient temperature difference; (**e**) luminosity; (**f**) vapor pressure deficit.

On the other hand, the temperature difference between the crop and leaf is taken as

(5) $$DT_{la}=T_{c}-T_{a}$$

where $T_{c}$, $T_{a}$ and *RH* are the leaf temperature, air temperature and relative humidity, respectively. Figure 11 shows the analysis of the information downloaded from Thingspeak and processed independently, showing the evolution during the 7 days for: (a) environmental temperature, (b) relative humidity in environment, (c) crop temperature, (d) difference in crop minus environment temperature, (e) luminosity and (f) vapor pressure deficit.

Table 4 describes the behavior of each variable with respect to the maximum, minimum, average during the 7 days and standard deviation, as well as the time in which the maximum and the minimum were determined for each case. Additionally, the behavior of pH, EC, fresh weight, length and leaf width were added.

**Figure 12:**
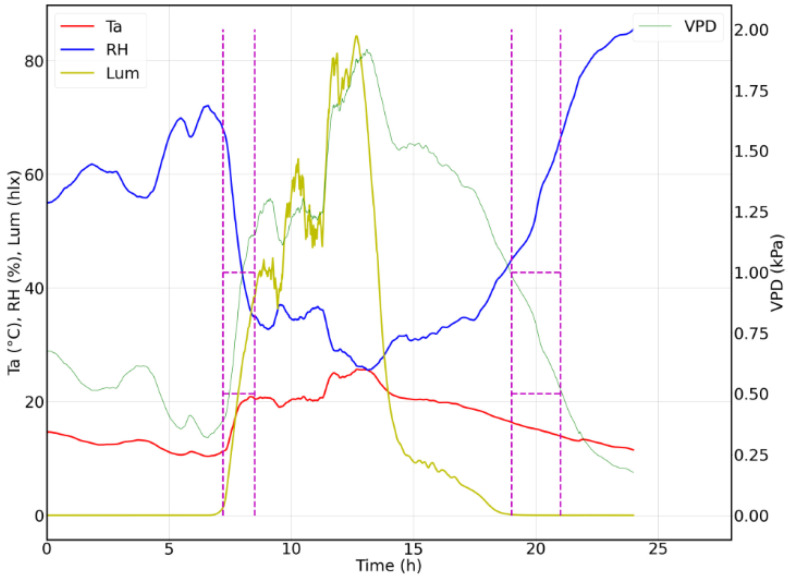
Favorable conditions for a low VPD.

Regarding the stress indicators, it is observed that the temperature of the leaf slightly exceeds 0 and the difference moves away to −4.63 °C when the luminosity is at its maximum point, approximately at 12 h, the same time in which the environmental temperature is higher than 25 °C and the VPD is above 2.5 kPa on most days. According to Amitrano, the crop tries to avoid dehydration and water loss by closing its stomata, reducing its phosphorus capacity and limiting the growth of the plant [20]. According to the information presented, the day with the lowest VPD was fuel day 3, on which, as shown in Figure 12, a VPD between 0.5 and 1 kPa developed in two hours: the first between 7:00 and 8:30 h when the temperature was between 10 and 20 °C and the relative humidity was between 35 and 70%; the second after 19:00 h, when there was practically no sunlight and the temperature dropped below 18 °C, while the humidity increased above 40%.

**Figure 13:**
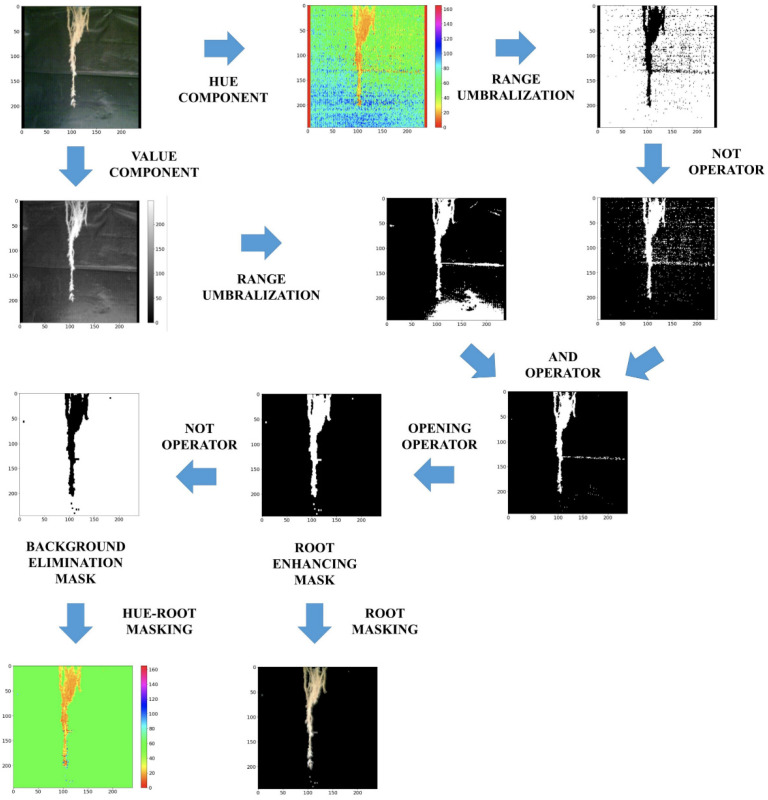
Image processing for root enhancement.

On the other hand, according to the images captured with the thermographic camera in the root (Figure 10), similarities were found in the colorimetry of the image seen from the thermal image, except for day 7, while in the RGB image, slight changes in colorimetry were found that were not so distinguishable after the first days. From this observation, a system for processing images to highlight the root was generated in the RGB and HSV model through the generation of a mask that only allows the root to pass and another to eliminate the background, respectively (Figure 13). The image processing is generated through the segmentation of the H and V components; for H the colors facing the background are segmented (35,140), while for V the pixels with high intensity are segmented (100, 255). Once a first mask is obtained, the AND operation is performed between the image segmented in V and the negative of that segmented in H to pass them through morphological filtering with a 2 × 2 kernel, obtaining from this process and its negative the masks for the root enhancement and background removal on the RGB and HSV images, respectively.

**Figure 14:**
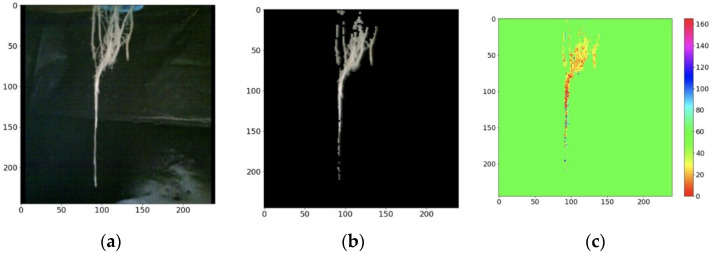
RGB and HSV images enhanced at root (day 1). (**a**) Original image of the root; (**b**) enhanced RGB image; (**c**) enhanced HSV image.

From the RGB image shown in Figure 10 and the processing described above, a tool was developed in the intelligent monitoring system to visualize the root with the enhanced image in the RGB and HSV model. The result of the masking of the image in each model is presented in Figure 14. 

**Figure 15:**
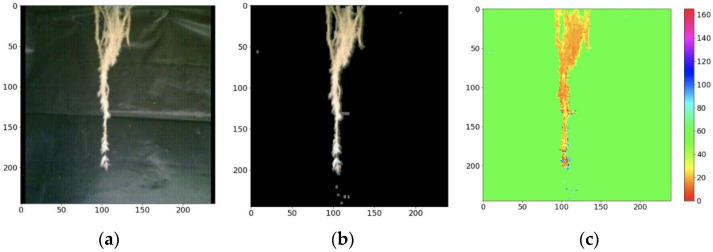
RGB and HSV images enhanced at root (day 7). (**a**) Original image of the root; (**b**) enhanced RGB image; (**c**) enhanced HSV image.

Comparing Figure 14 and Figure 15, the change in tone of the root can be seen darkening due to water stress. The darkening of the root is distinguished by the gradual increase in reddish tones in the root seen in the HSV model.

**Figure 16:**
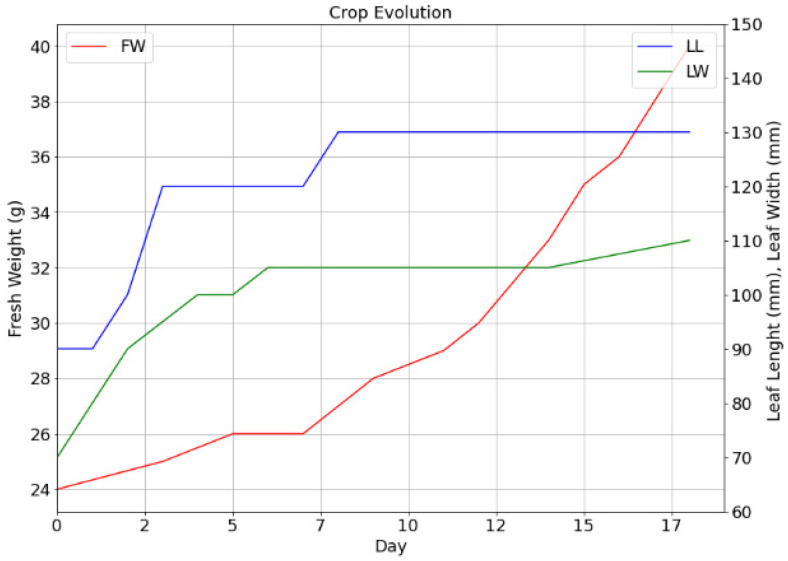
Yield evolution regarding fresh weight, leaf length and width in the test plant.

Relating to the evolution of the yielding of the crop referring to the fresh weight, and the length and the width of the leaf, the measurements were made using a scale and a standard flexometer. The data were stored on the Thingspeak server via the application developed in this study. Figure 16 shows a continuous increase in the fresh weight of the crop until reaching a gain of 16 g on day 18 from its transplant to the aeroponic chamber; likewise the growth of the leaf initially increased by 30 mm in the first days and later achieved a minimum growth from day 7 to 18.

## 5. Discussion

Various studies have indicated that decision making to generate the conditions of irrigation in IoT architectures take as reference the evapotranspiration, the weather forecast, the relative humidity, the irrigation according to the life cycle and the irrigation programmed by the user in soil, while in a crop without soil, the variables considered for changing the irrigation scheme are the temperature and relative humidity in the environment, and the programmed irrigation schemes based on frequency and the ignition time. Particularly in aeroponics, between the schemes recently used are the variation in atomization times of day/night according to the days of production and the time interval when the ambient temperature exceeds 35 °C [14], as well as the control of the temperature and humidity in the root chamber between 25 and 29 °C [40]; however, they do not consider the standard markers of crop water needs such as the crop temperature, VPD or water stress index. 

In the present paper, a Batavia lettuce seedling was subjected to observation in the aeroponic cultivation chamber from its transplant, preserving the ranges of pH and EC between 5.5 and 6.5 and 750 and 1100 ppm and with a constant irrigation system every 24 min day/night with an activation time of 30 s. According to the experimental results, the DT_la_ does not provide a specific criterion for the determination of water stress in an aeroponic crop; more studies are required on the Batavia lettuce variety to estimate the temperature ranges where the DT_la_ serve as a concrete marker of water stress. The experimentation in the crop camera that was presented shows how the VPD from the beginning marked a potential root damage due to water stress. Figure 9 illustrates how a temperature between 10 and 20 °C together with a relative humidity above 35% favors the reduction in the VPD. Figure 16 shows, in relation to the fresh weight, an increase in VPD reduction. Figure 16 shows, in relation to the fresh weight, a consistent increase; however, the leaf size presented a reduction in the rate of growth from the first week.

The estimation of the VPD in conjunction with image processing provides an estimate of the appearance and evolution of water stress in aeroponic crops. Among the main actions to evaluate against a high VPD and the reduction in the rate of growth of the leaf, is the increase in the frequency of irrigation, as well as the incorporation of a system that allows the control of external environmental parameters.

In the literature studied, both conceptual and practices in the development of crops in the soil, hydroponics and aeroponics, address the grouping schemes of components that make up the stages and connectivity of an IoT architecture. 

According to the practical proposals found in the literature, by using IoT-based technologies to manage irrigation conditions and proposals related to aeroponics, in comparison with our proposal, the following were found: similarities between layers using IoT architecture and functionalities using functional blocks addressed by Boursianis, Roy and Lloret; common study variables addressed by Lucero, Jamhari and Belista. According to this criterion, the differences are highlighted in Table 5.

As shown, most of the systems consider the measurement of environmental variables and, depending on the meteorological data of the case to determine the irrigation and monitoring needs, the automated monitoring of the nutritional conditions of the nutritive solution. However, the previous studies do not consider the monitoring of crop growth from image acquisition and analysis systems, nor do they include reports and alerts due to the failure of the electronic systems used in the proposal. In the proposal that is presented in this paper, there is an application that not only accesses the monitored data regarding the crop, but also provides information on the functionality status of the incorporated electronic systems, allowing the user to be alert to the maintenance needs of electronic elements and software incorporated in the IoT architecture that has been presented. This proposal does not yet incorporate the temperature control and humidity in the greenhouse; however, the architecture developed presents the flexibility to incorporate additional monitoring services and control due to the inclusion of a fog node in the proposed IoT architecture.

## 6. Conclusions and Further Studies

This article presents an IoT architecture that facilitates the management of an aeroponic greenhouse through the registration of manual procedures, the automation of the irrigation system and the monitoring of environmental variables in cultivation; all this with the support of a visualization tool from an app. 

The estimation of the VPD indicates the appearance of water stress due to the insufficient absorption of crop water. This suggests the importance of making an adaptation to the atomization times and the intervals of the times of irrigation, as well as the cooling and ventilation systems.

The main contribution of this paper is the integration of smart sensors, in a technological solution based on IoT, that allows the study of the favorable conditions for the growth of aeroponic crops with remote management. The app developed for this purpose allows the visualization of historical variables of temperature and relative humidity in the environment and crops, VPD and luminosity, as well as the level and temperature of the nutrient solution tanks to carry out the adjustment of pH and EC. The proposed architecture allows the incorporation of monitoring and image processing systems that will enrich the studies of the thermography and morphology of the aeroponic crops for roots and leaves. Additionally, the proposed architecture manages the heterogeneity of data through microservices and allows us to reduce latency in the communication with the server using, as a basis, a waiting interval time of 30 s between each writing of data on the server, as well as by using a reduced bandwidth to view the results of image processing remotely, by a request to the server and downloading it from the app.

The use of the proposed architecture will allow the study of the variation in irrigation schemes based on markers such as VPD, CWSI and DT_la_, favoring the rapid adaptation of irrigation strategies to produce crops with a higher number of leaves, leaf area index and fresh weight among others. 

As future work, the proposed architecture for aeroponic crop production will be implemented, monitoring the growth chambers simultaneously, and considering the irrigation frequency and the time interval as study variables, as well as the decrease in VPD in the aeroponic crop process. With regard to the image processing-embedded algorithms on fog layer, for the study of colorimetry, infrared thermography and morphology in the evolution of the crop when considering the leaf and root will be added.

## Figures and Tables

**Table 1:** Notation and nomenclature used for this article.

Symbol	Meaning
T_a_ (°C)	Ambient temperature
RH_a_ (%)	Ambient relative humidity
T_c_ (°C)	Crop temperature
RH_c_ (%)	Crop relative humidity
Lum (lux)	Ambient light intensity
T_sol_ (°C)	Nutrient solution temperature
L_sol_ (l)	Nutrient solution level
T_res_ (°C)	Recirculating tank temperature
L_res_ (l)	Recirculating tank level
S_irr_ (on/off)	Irrigation pump status
S_res_ (on/off)	Recirculation pump status
S_cam_	Image acquisition system status
pH	Hydrogen potential
EC (µS/cm)	Electroconductivity
TDS (ppm)	Total dissolved solids in the nutrient solution
Fw (gr)	Fresh weight
L_l_ (mm)	Leaf length
W_l_ (mm)	Leaf width
HNO_3_ (mL/L)	Nitric acid
A_sol_ (mL/L)	Solution A
B_sol_ (mL/L)	Solution B
DT_la_ (°C)	Difference Temperature
VPD (kPa)	Vapor pressure deficit

**Table 2:** Comparison considering elements included for monitoring, control and irrigation.

Author/Architecture	Year	Objective/ Reference	Architecture/Sensors/Data Base/Protocols
Kamiienski et al./SWAMP [32]	2018	Soil	Five-layer architecture (application, distribution, data management, acquisition and security, communication)
Filev et al./ IRRISENS [34]	2020	Soil/Relative Humidity	Relative humidity, climatic conditions, local/cloud DB.
Boursianis et al./AREThOU5A [35]	2020	Soil	Five-layer architecture (physics, data link, network, authentication, application). Temperature and humidity, local/cloud DB, LoRaWAN, TCP/I, MQT, SL. Solar battery charging.
Roy et al./AgriSens [36]	2021	Soil/Life Cycle	Three-layer architecture (sensors and actuators, remote server, application). Humidity. Level. DB cloud. ZigBee, GSM/GPRS.
González-Amarillo et al. [37]	2018	Germination	Temperature, humidity, luminosity, water consumption. Local/cloud database.
Fernández-Ahumada et al. [38]	2019	Soil/Relative Humidity	Three-layer architecture (sensors and actuators, application, final user). Relative humidity. LoRa, SigFox, Thingspeak.
Mohammed et al./CSIS [39]	2021	Soil/ET	Volumetric water content, relative humidity, temperature, solar radiation, speed of wind, flow.
Poyen et al./SAIC [40]	2021	Soil/ET	Air/soil temperature, relative humidity, wind speed, solar radiation, atmospheric pressure. Local/Cloud DB. GSM/GPRS.
Lloret et al. [41]	2021	Flood/User	Perception, service, application, end user. Relative humidity, temperature, atmospheric pressure, rain. Temperature, salinity, level, water pests. HTTP.
Lucero et al. [14]	2020	Aeroponics/ Temperature, Relative Humidity	Temperature, relative humidity, level, pH. GSM/GPRS
Gour et al. [43]	2020	Aeroponics	Two-layer architecture (sensors and actuators, services). Relative humidity, temperature, CO_2_, pH, luminosity. Machine learning.
Belista et al. [44]	2018	Aeroponics	Three-layer architecture (sensors and controllers, data storage and processing, application platform). Temperature, relative humidity, level of water, EC, pH. Local DB
Our Proposal	2022	Aeroponics/VPD, Irrigation Period	Four-layer architecture (device, fog cap, cloud, application). Leaf temperature, environmental temperature and relative humidity, luminosity, pH, EC, level and nutrient solution temperature, RGB and thermographic images. Warning against service failure, status of sensors and actuators. HTTP. IoT analytics, Thingspeak, Firebase.

**Table 3:** Variable—Report Relationship using MATLAB Analysis and Visualization.

	Ambient Measurement	Crop Measurement	Difference Temperature	Luminosity	Solution Measurement	Reservoir	Inspection	VPD	Solution Parameters	Crop Parameters	Request per Hour	Request per Channel
T_a_	X		X					X			X	X
RH_a_	X										X	X
T_c_		X	X								X	X
RH_c_		X						X			X	X
Lum				X							X	X
T_sol_					X						X	X
L_sol_											X	X
T_res_					X						X	X
L_res_						X					X	X
S_irr_						X					X	X
S_res_						X					X	X
S_cam_							X				X	X
pH									X		X	X
EC									X		X	X
Fw										X	X	X
L_l_										X	X	X
W_l_										X	X	X
HNO_3_									X		X	X
A_sol_									X		X	X
B_sol_									X		X	X

**Table 4:** Analysis of main variables in the proposed intelligent monitoring system.

Variable	Minimum	Maximum	Mean	Std	Time (max)	Time (min)
T_a_	6.47	34.19	17.91	6.90	12:52	07:01
T_c_	5.87	30.66	16.72	5.93	13:11	07:02
RH_a_	5.87	97.43	37.78	21.24	07:02	10:40
RH_c_	12.45	83.62	42.13	15.68	07:20	12:27
Lum	0	9118	1702	2697	12:23	-
DT_la_	−4.63	0.24	−1.19	1.19	00:00	11:52
VPD	0.014	3.766	1.338	0.8526	12:25	07:01
pH	5.76	6.44	6.08	0.1859	-	-
EC	783	1084	907.6	69.78	-	-
Fw	24	26	24.85	0.8329	-	-
L_l_	100	135	127.1	11.29	-	-
W_l_	70	85	127.1	127.14	-	-

**Table 5:** Comparison with related studies.

Features			Own	Lucero [14]	Boursianis [33]	Roy [34]	Lloret [39]	Jamhari [40]	Belista [42]
Sensor Nodes	Ambient		X	X	X	X	X	X	X
	Ambient in Crop		X						
	VPD Estimation		X						
	RGB Image		X						
	Thermographic Image		X						
	Meteorological				X		X		
	Nutrient Solution		X	X					
Alert System			X	X			X		
Programmable			X			X	X		
Automatic Control		Temperature		X				X	
		Humidity						X	
		pH							
Friendly Interface	Remote Monitoring	App	X	X			X		X
		VPN	X						
		GSM		X	X	X			
		Web Server	X	X		X		X	
	Sensors Status		X						
	Actuators Status		X						
Applicable for crop diversity			X		X	X			

## Data Availability

The data presented in this study are available on request from the corresponding author.
